# Reducing sickness absence: a work environment intervention in Norwegian hospitals

**DOI:** 10.1186/s12913-024-11373-6

**Published:** 2024-08-12

**Authors:** Andreas Lillebråten, Anne Grete Tøge, Vilde Hoff Bernstrøm

**Affiliations:** Work Research Institute, Oslo Metropolitan University, Oslo, Norway

**Keywords:** Intervention, Tailoring, Work environment, Sickness absence, Health, Job satisfaction, Healthcare, Hospitals, Propensity score matching

## Abstract

**Background:**

High rates of sickness absence is a challenge within the healthcare sector, highlighting the need for effective interventions. Despite this, limited research has been conducted on the impact of such interventions within the healthcare context. This study evaluates an intervention aimed at improving the work environment influences sickness absence rates in Norwegian hospital units. The intervention is a comprehensive framework for discovering and tailoring solutions to each units’ specific needs, with a focus on employee involvement and collaboration between leader, union representatives and safety delegates.

**Methods:**

We employed two methodological approaches. Method 1 involved using HR-registered sickness absence data to track changes in sickness absence across all intervention units and matched control groups over a three-year period. In Method 2, we used a pre- and post-survey design in 14 intervention units, focusing on employees’ job satisfaction and self-reported health.

**Results:**

The results of the intervention were mixed. There was a significant decrease in total sickness absence in the intervention units the first year after the intervention, and a significant decrease in long-term sickness absence both in the first and second year after the intervention, measured with HR registries. However, we did not see a significant larger decrease in total sickness absence in the intervention units compared to the control units and only partial support for a larger decrease in long-term absence in the intervention units. In the subsample of units that also participated in the survey, we observed significant improvements in employee job satisfaction post intervention.

**Conclusions:**

There is a need for research on effective interventions to reduce sickness absence in the healthcare sector. “Where the shoe pinches” provides a potential methodological framework for reducing sickness absence by addressing challenges in the work environment, however with uncertain results. Further exploration is warranted to refine strategies for effectively managing sickness absence within healthcare organizations.

## Introduction

The healthcare sector faces a challenge with high rates of sickness absence [1]. Consequently, it is crucial to identify and implement interventions that effectively reduce sickness absence in the healthcare sector. Previous reviews of the literature indicate that certain interventions can effectively reduce sickness absence. Interventions vary widely, encompassing physical activity [2, 3], health promotion [4], and efforts to improve the psychosocial work environment [5]. While several studies explore how interventions affect sickness absence in the workplace in general [6], there are few done on employees in the health care industry. Arguably, interventions from other sectors may not translate to the health care industry due to the uniqueness of the sector. The healthcare industry stands out from other sectors due to its long, unpredictable hours, high emotional demands, and limited job control [7]. Simmons et al. [8] concluded in their review that existing interventions in healthcare sector fail to effectively reduce sickness absence. Only a few interventions studies have been published since the review, supporting positive effects of exercise [9] and meditation [10].

Focusing on interventions targeting the work environment could be promising for reducing sickness absence. Prior studies have supported that both intervention targeting the physical, organizational and psychological work environment may be effective in reducing sickness absence [5, 11, 12]. However, while the importance of the work environment was emphasized by Brady et al. [13], who identified it as a crucial determinant of sickness absence within the healthcare sector, there is a lack of studies exploring this type of intervention within the health care industry. The limited research available, such as Cedstrand et al. [14] and Kester et al. [15], reports varied impacts on employee health and wellbeing.

Therefore, our study investigates how an intervention aimed at improving the work environment affects sickness absence rates within the healthcare sector.

### Tailoring as an intervention

The name of the intervention is “Where the shoe pinches”, an idiomatic expression used to describe the root cause of a problem. The intervention is designed to first identify the underlying root causes of challenges in the work environment. Subsequently, it develops and implements customized measures to address these challenges, with ongoing revaluation and adjustment. Its primary objective is to improve the work environment, thereby reducing and preventing sickness absence and promoting employee health and well-being.

In essence the intervention is thus a framework to identify and address challenges tailored to the specific unit. Tailoring has been defined as “any combination of information or change strategies intended to reach one specific person, based on characteristics that are unique to that person, related to the outcome of interest, and have been derived from an individual assessment” [16, 17]. In the case of “Where the shoe pinches” the tailoring is a change strategy intended to reach one specific hospital unit, based on characteristics that are unique to that unit, related to the work environment and sickness absence, and which have been derived from an individual assessment of the hospital unit. The intervention “Where the shoe pinches” is thus a framework design to facilitate the assessment of the hospital unit and the selection and execution of the tailored change strategies. Importantly, “Where the shoe pinches” thus deviates from a traditional definition of tailoring by focusing on the hospital unit, rather than the individual.

Over the past decade, numerous studies have explored tailored interventions across various fields [18]. Several of these studies focus on health education interventions [19, 20], but also include student absence interventions [21], family interventions [22], and interventions to enhance collaboration [23]. There has also been support that both tailoring and targeted intervention may be more effective than standard interventions [24, 25]. This increased effectiveness can be attributed to the fact that tailored and targeted interventions take into account the specific characteristics of the recipients [26, 27].

### The intervention: “where the shoe pinches”

The intervention consists of several distinct yet interconnected stages and activities. Three key components of the intervention are (1) a strong focus on collaboration between the leader, the union representative, and the safety representative throughout the process, (2) a process facilitator from outside the unit, and (3) the “dialog cloth”. Units are selected to participate based on a history of prolonged high absence rates. For a more thorough description of the intervention see Fjeldbraaten and Wathne [28].

### Who – the process facilitator, leaders, union representatives, and safety delegates

A process facilitator oversees the intervention. Process facilitators usually belong to the hospital’s HR department, work environment department, or company health service. Furthermore, advisors from the Labour and Welfare Administration may also serve as facilitators. Process facilitators should have expertise in process management and of health, safety, and work environment practices. The process facilitator should be familiar with the hospital’s systems for health, safety, quality, and patient safety, integrating the methodology into existing routines and systems.

The process facilitator is responsible for facilitating a structured collaboration between unit leaders, union representatives, and safety delegates. This collaboration is central throughout the intervention.

While the process facilitator plays a crucial role in the intervention, ultimate ownership and overarching responsibility for implementation lie with the unit leader. Moreover, successful intervention necessitates collaboration among the leader, the union representative, and the safety representative. Regular cooperation and evaluation meetings among these parties are integral components of the intervention process.

### Materials - the “dialogue cloth”

The “dialogue cloth,” a tablecloth designed for dialog facilitation, is used during a workshop lasting approximately three hours. The tool is used in stage 2 for employees (see stage 2). Comprising various assignments, the dialogue cloth requires groups of 3–5 employees to engage. Some tasks are individual, while others demand group collaboration, all aimed at fostering discussions and reflections on the work environment. For example, two assignments prompt individuals to contemplate “What works well for you in your job?” and “What should be improved?” Participants first document their thoughts individually before engaging in group discussions. These workshops are facilitated by the process facilitator, who also holds the responsibility of summarizing workshop outcomes. A modified dialogue cloth was implemented in stage 1 in April 2022.

### Process – the four stages

The process consists of four interlinked stages.

***Stage 1 Preparation and Planning*** In this initial stage of the intervention, the process facilitator take responsibility for planning, with the aim of crafting a customized intervention plan tailored to the specific unit’s needs. The process facilitator gathers information about the unit’s unique requirements, considering factors like size, staff numbers, challenges, and time constraints. Additionally, the process facilitator is tasked with engaging leaders, union representatives, and safety delegates through dialog to ensure their active participation in the intervention process. However, it was observed during the intervention period that collaboration between the parties often did not meet the intended objectives. Starting from April/May 2022, leaders, union representatives, and safety delegates from a new unit participated in a dialog workshop using a customized dialog cloth. This cloth was specifically tailored to address their understanding of a constructive work environment, promote cooperation, enhance communication, and clarify different roles.

***Stage 2 Mapping***, ***Analysis***, *** and employee involvement*** The second stage of the intervention is centred on conducting a comprehensive assessment of the work environment, using both existing data and targeted new assessments facilitated and summarized by the process facilitator. Initially, a two-hour assessment conversation is held with the unit leader, covering topics such as the work environment, leadership perceptions, team cooperation, personnel issues, and other relevant areas. Similarly, assessment conversations are conducted with union representatives and safety delegates to gauge the employees’ perspectives on the work environment. These may take the form of either individual interview (lasting 1.5 h), or as a group interview (2 h), addressing topics such as the work environment, partnership perceptions, leadership, personal challenges in critical areas, and other relevant issues. Subsequently, employees are engaged through a **dialog workshop**, using group discussions facilitated by the dialogue cloth. In a few instances were the unit had personnel conflicts not suited for group discussion the dialog workshop was replaced by individual conversations. The aim is to identify strengths and areas requiring improvement within the work enviorment. Stage two culminates in an analysis of the work environment integrating findings from all the above-mentioned methods to identify challenges and strengths. The process facilitator compiles this analysis into a report, offering a roadmap for targeted improvements and ensuring that the most pressing issues are addressed.

A summary of collected reports from the process facilitators showed that identified challenges included challenges with physical environment, the organizational work environment, and the psychosocial work environment, as well as quality of patient treatment. Most units reported multiple challenges within all four areas. Within the physical work environment example of challenges included indoor climate, equipment, noise, ergonomics, and lack of order. Within the organizational work environment example of challenges included staffing and workload, distribution of tasks, management, work hours, information and employee participation. Within the psychosocial work environment examples of challenges included collaboration and communication, conflict and bullying, respect, and support. Within the area of patient treatment professional development and training, improvements of and adherence to routines and rules.

***Stage 3*Implementation** In this third stage of the intervention, employees and key stakeholders collaborate to develop and implement measures aimed at addressing the challenges identified in stage two.

The report from stage two is presented to the employees by either the leader of the unit, a union representative, or the safety delegate, with the support of the process facilitator. Employees are then organized into groups to discuss possible measures or addressing the identified areas of improvement outlined in the report. The ideas generated during these sessions are to be summarized, often by the process facilitator, although the responsibility is not explicitly assigned.

Once the summary is prepared, leaders, union representatives, and safety delegates assume responsibility for operationalizing and prioritizing the measures. The process facilitator serves as an advisor, offering insight into which measures are expected to be most effective. The prioritized measures are subsequently formulated into a strategy with deadlines and assigned responsibilities.

The actual measured implemented varied between the units. Some measures were intended to be preventive while others were focused on solving existing problems. Measures focused on the individual level as well as the unit level. Some measures were simple while others involved complex processes. In one unit where physicians struggled with musculoskeletal pains due to prolonged static, workflow was rearranged so that the physicians took turns relieving each so no one was left standing still for too long at a time. Other examples measures included reducing noise, purchasing ergonomic equipment, participating in courses, upgrading social areas, and facilitate professional discussions [28].

***Stage 4 Evaluation and Adjustment*** The fourth and final stage concentrates on follow-up, evaluation, and adjustment of implemented measures. Leaders, union representatives, and safety delegates hold regular meetings to evaluate the progress of prioritized and implemented measures, actively involving employees in this evaluation process. The strategy undergoes regular updated based on this continuous feedback loop, ensuring a dynamic approach to quality improvement within the unit.

### Method

The intervention was completed in a total of 78 hospital units, with 42 units in 2021 and 36 units in 2022. The first unit initiated the intervention in January 2021, and the last in December 2022. We included units from all four Norwegian regional health trusts, which varied in size and employee profession.

To examine the intervention’s outcomes, we employed two methodological approaches. Method 1 involved using HR-registered sickness absence data to track changes in sickness absence across all intervention units and matched control groups over a three-year period. In Method 2, we used a pre- and post-survey design in 14 intervention units, focusing on employees’ job satisfaction and self-reported health. In both methodological approaches, we consider the month of the **Dialog workshop** as the starting point of the intervention.

### Method 1 h registered sickness absence

#### Data

Each Regional Health Trust provided monthly sickness absence data per unit for each hospital from 2020 to 2022. This resulted in a dataset comprising sickness absence records from all units within Norway’s public hospitals.

Of the seventy-eight units participated in the intervention we monitored each unit for 14–35 months prior to the intervention and 1–23 months afterward (depending on the intervention start date).

The remaining hospital units (i.e. who did not participate in the intervention) were used as a pool for selecting a control group (se analyses). Following consultations with the health trusts about unrealistic values, we excluded units lacking more than one registered employee, those with negative absence figures, or those missing a unitID number. Finally, we excluded all units without complete data in 2020. In total 5135 units remained as potential control units. Additionally, 6 intervention units also lacked complete data for 2020 and were thus excluded from all analyses with control groups.

#### Variables

The data comprised information on sickness absence, the intervention, and the size of the unit. Intervention: Regarding the intervention, all months before its initiation were coded as 0. We designated the month of the dialogue workshop and the following 11 months as the first year of the intervention, coded as 1. All subsequent months were coded as 2, representing the second year of the intervention.

Sickness absence was quantified as the unit’s percentage of total absence days ((number of absence days / person-month in the unit) * 100) and the unit’s percentage of long-term absence days ((number of absence days in spells lasting 17 days or longer / person-month in the unit) * 100). The threshold for long-term absence spells was set at 17 days. In Norway, this duration marks the point where the absent employee’s salary is funded by the state rather than the employer [29]. Notably, there were variations among the regional health trusts in coding the number of absence days. Some trusts recorded a day as an absence day only if the employee was scheduled to work on that day (e.g., if a part-time employee worked 3 days a week, a doctor-certified sick leave of 14 days was recorded as absence for only 6 days). This discrepancy in how absence was coded between the health trusts led to regional differences in absence rates, and for some trusts, lower absence rates than comparable official statistics.

Unit size was measured in person-months at the unit.

#### Analyses

We conducted two groups of analyses, fixed effects and multilevel modelling with propensity score matching. In all analyses of sickness absence from HR registries we used a Poisson regression to account for the non-normal distribution of count data, the coefficient is thus the log of the expected count. The count being the unit’s percentage of total and long-term absence days.

We used fixed effects analyses to investigate the development of sickness absence within the intervention units. In a fixed effects analyses each intervention unit is only compared to itself; we only analyse variation in absence within the units over time. In these analyses each unit serves as its own control – comparing monthly sickness absence in the first and the second year of the intervention to the same unit’s absence from January 2020 until the month prior to the intervention. In this manner stable differences between units are controlled for by the model. Year and quarter were included as a control to account for fluctuation in absence rates due to season and COVID-19 restrictions.

We then used a multilevel modelling to investigate the development of sickness absence within the intervention units compared to a control group. We analysed if any changes from prior to the intervention to the first and second year after the intervention were significantly different between the intervention and control units.

We used multilevel modelling with a random intercept at unit and regional health trust-levels, allowing observations to be nested within units, and units to be nested within their regional health trusts. By allowing each unit and health trust to have their own random intercept the model accounts for dependency in the data and stable differences between units and health trusts.

Units participating in the intervention were selected due to prolonged high sickness absence. Consequently, it is anticipated that there would be a reduction in sickness absence over time regardless of whether the intervention is implemented (i.e. regression towards the mean). To handle this bias we employed propensity score matching to select a control group for the multilevel modelling. Propensity score matching enabled us to create a control group comprising of up to five units matched to each intervention unit. The control units had not implemented the intervention but were similar to those in the intervention group, including similar absence levels in 2020 pre-intervention. We use data from all units in 2020 (prior to the first intervention) to calculate a propensity score. The propensity score is a calculated probability of a unit participating in the intervention, conditional on pretreatment covariates [30, 31]. The confounders are covariates that affect outcomes and treatment assignments. As confounders we included total absence and long-term absence for each month in 2020, regional health trust, and size the first and last month in 2020, expecting all to be relevant for the selection of units to the intervention. We used the psmatch2, logit command in STATA. And in line with [32] we specified caliper distance as 0.2 of the pooled standard deviation of the logit of the propensity score. The caliper distance specifies the maximal acceptable distance between the intervention and their selected neighbors. The mean propensity score and standard deviation were low (mean 0.014 and std. 0.02) – and the caliper were therefore set to 0.004.

We used matching with replacement, which implies that for each intervention unit matched controls are selected from all potential control variables. Consequently, some control units are matched with more than one intervention unit. Due to the complicated nesting structure in the data, we dropped duplicate control units from prior to the final analyses, allowing each control unit to be matched with only one intervention unit.

All units were given a temporal variable (i.e. 0 “baseline”, 1 “first year post intervention”, 2 “second year post intervention”). Control units were given a value identical to the intervention unit it was matched with. If intervention unit A implemented the intervention in January 2021, each of the five control units matched with unit A were also given the value baseline for the time prior to January 2021, and 1 “first year post intervention” and 2 “second year post intervention” for the years following. In this manner time trends, such as seasonal differences or changes in COVID related challenges, are kept constant between the intervention units and each intervention unit’s matched control units. In this manner, the method also accounts for the intervention being implemented at multiple time points during 2021 and 2022.

No control variables were included in the analyses, as all potential control variables were used in the propensity score matching.

### Method 2 longitudinal pre-post survey

#### Data

Fourteen Norwegian hospitals units participated in the longitudinal survey. The units were recruited by the intervention organizers based on the timing of the intervention implementation. These units are spread across all four of Norway’s health trusts, spanning the country geographically. Unit sizes ranged from 14 to 89 employees.

We conducted three surveys: the pre-intervention survey (T1), administered two weeks prior to the dialog workshop, the process-survey, administered two to seven days after the intervention was started (T2), and the post-intervention survey (T3), conducted one year later in 8 of the units. Due to time constraints, the remaining 6 units received T3 between 9 and 11 months after the first dialog workshop. T1 and T2 were distributed from September 2021 to June 2023, while T3 was administered from September 2022 to March 2023. The T1 and T3 survey measured changes in key variables pre and post intervention (i.e. absence, general health, and job satisfaction). The T2 survey focused on the implementation of the intervention.

In T1, a total of 283 out of 643 employees responded our survey, yielding a 44% response rate, with individual unit response rates varying between 20% and 69. In the T2 survey, conducted after the intervention, 242 employees responded, achieving a 38% response rate, with unit response rates between 22% and 71%. In T3, 197 employees responded, resulting in a 31% response rate, with unit response rates varying from 13 to 79%. Fifty-eight employees responded at both T1 and T3.

#### Variables

### Intervention

Our explanatory variable is whether the intervention is implemented or not, with time before implementation coded as 0 and after coded as 1.

### General health

We assessed general health at T1 and T3 using a scale derived from 5 variables in the 36-Item Short Form Survey Instrument (SF-36) [33]. First, we asked respondents, “In general, would you say your health is.” with five categories ranging from 1 “excellent” to 5 “poor”. Further, we asked, “How TRUE or FALSE is each of the following statements for you.”, followed by four statements: “I seem to get sick a little easier than other people”, “I am as healthy as anybody I know”, “I expect my health to get worse” and “My health is excellent”. The respondents could answer on a scale from 1 “Definitely true” to 5 “Definitely false”. We recoded the scale to range from 0 to 100, with higher scores indicating better health.

### Job satisfaction

Job satisfaction was measured at T1 and T3 using three items from The Michigan Organizational Assessment Questionnaire (MOAQ) [34]: ‘‘All in all, I am satisfied with my job.”, ‘‘In general, I don’t like my job.” and ‘‘In general, I like working here.”. Respondent rated their agreement on a seven-point scale ranging from 1 “Strongly disagree” to 7 “Strongly agree”.

### Sickness absence

We measured sickness absence and long-term sickness absence at T1 and T3 as two dichotomous variables. Sickness absence was determined by the response the item “Have you been absent from work due to your own illness in the last 6 months?”. Responses was coded 1 for “Yes” and 0 for “No”. Long-term sickness absence was gauged using the item “How many consecutive days did the absence(s) last?” Responses of “8 days or less” and “between 8 and 16 days” were coded as 0, and “17 days or more” was coded as 1.

### Control variables

We control for age, sex and whether the respondent worked evening shifts or night shift.

### Intervention process

In the process-survey (T2) we measure five aspects of the implementation process, central in the intervention: *unit leader*,* union representative and safety delegates involvement in the intervention*,* employee involvement*,* and collaboration within the intervention workgroup*. While the first four aspects were measured among all employees, the fifth —collaboration within the workgroup—was specifically directed at workgroup members only.

The *unit leaders involvement in the intervention*, was measured by 6 items adapted of Randall et al. [35] Line manager attitudes and action (e.g. “My immediate manager was positive about the implementation of teams” was altered to “My immediate manager is positive towards “were the show pinches”.

*The union representative and safety delegate involvement* was measured using an adaptation of three of the same items from the same scales “(e.g. My union representative is positive towards “were the show pinches”). Response ranged from 1 “Strongly Disagree” to 5 “Strongly agree”.

*Employee involvement* in designing and implementing the intervention was measured with three questions from Randall et al. [35]. An example question is: “Management has made a great effort to involve employees in the change process”. Response ranged from 1 “Strongly Disagree” to 7 “Strongly agree”.

The scale for *collaboration within the intervention workgroup* (i.e. c*ollaboration between the leader*,* the union representative and safety delegates)* was designed to capture collaboration between the individual represented different roles while being asked to collaborate as a team or work group for the intervention. The scale consists of eight items (e.g, “in the work group, we agree on what our most important tasks are” and “The others in the workgroup are willing to discuss my suggestions for change”). The items were inspired by and adapted from the social capital team scale, social capital inter-team scale (social capital between teams) [36], and interprofessional collaboration [37]. Response ranged from 1 “Strongly Disagree” to 5 “Strongly agree”. Only the leader, union representative and safety delegates answered the questions pertaining to their collaboration.

In the post-intervention survey (T3), we also constructed a question to assess *how much focus was placed on the intervention*: “Your team started working on ‘Where the Shoe Pinches’ with a dialogue workshop on [DATE]. From then until now, how much focus have your team placed on the work with ‘Where the Shoe Pinches’?“, with responses ranged from 1 ”Very little” to 5 “Very much”.

#### Analyses

As our dataset comprised employees from various hospital units, it inherently possessed a hierarchical structure, leading to dependency among employees within the same hospital unit. To account for this hierarchical data structure and the associated dependencies, we employed a multilevel model. This model included random intercepts for employees to capture individual variability and for hospital units to address the between-unit variance. This approach enables us to account for both the within-hospital unit correlation among employees and the differences between units. Our analysis employed multilevel linear regression to analyse continuous outcomes job satisfaction and general health, and multilevel logistic regression for binary outcomes related to sickness absence.

## Results

### Method 1 h registered sickness absence

Table 1 presents the results from the fixed effect models, comparing each unit with its own performance before and after the intervention. In Model 1, we observe a significant decrease in overall sickness absence during the first year following the intervention (-0.07, *p* < 0.001), but this correlation is not significant in the second year. Upon introducing control variables in Model 2, there is a significant decrease in both the first- and second-years post-intervention (T1: -0.14. *p* < 0.001 T2: -0.12 *p* < 0.01). Model 3 assesses the intervention’s effect on long-term sickness absence (17 + days), revealing a significant reduction in absence in both the first and second years after the intervention (T1: -0.18, *p* < 0.001 T2: -0.13 *p* < 0.001). These results persist after including control variables in model 4 (T1: -0.20, *p* < 0.001 T2: -0.15 *p* < 0.001).

**Table 1:** Fixed effect poisson regression

	All sickness absence								Long-term sickness absence (≥17 days)							
	M1				M2				M3				M4			
Pre Intervention	ref				ref				ref				ref			
First year post Intervention	-0,07	***	(-0,10	-0,03)	-0,14	***	(-0,19	-0,09)	-0,18	***	(-0,23	-0,14)	-0,20	***	(-0,26	-0,14)
Second year post Intervention	0,00		(-0,06	0,05)	-0,12	**	(-0,20	-0,04)	-0,13	***	(-0,20	-0,06)	-0,15	**	(-0,25	-0,05)
Year																
2021					0,02		(-0,02	0,05)					0,03		(-0,02	0,07)
2022					0,12	***	(0,07	0,18)					0,05		(-0,02	0,11)
Quater																
2					-0,06	**	(-0,10	-0,02)					-0,02		(-0,07	0,02)
3					-0,25	***	(-0,29	-0,21)					-0,15	***	(-0,19	-0,10)
4					-0,09	***	(-0,13	-0,05)					-0,10	***	(-0,15	-0,05)

N: 2599/2635 observation from 75/76 units. Each unit included on average 35 months. * = *p* < 0.05, ** = *p* < 0.01, *** = *p* < 0.001

In the control variables, we observe a general increase in sickness absence during the study period. Repeating the fixed effects analyses for all hospital units with only year and quarter (not shown) show a general increase in sickness absence during the study period.

Table 2 presents the difference in total and long-term absence between the intervention units and matched control units in 2020 (prior to the intervention).We found no significant differences between the intervention units and the matched control units on long-term sickness absence. However, the intervention units exhibited significant lower total sickness absence after controlling for the nesting structure of the data in a multilevel model (coef = − 0.25, *p* > 0.01).

**Table 2:** Sickness absence in 2020 for intervention units and matched control units

	All sickness absence				Long-term sickness absence (≥17 days)			
	M5		95% CI		M6		95% CI	
Matched control unit	ref.				ref.			
Intervention unit	-0,26	**	(-0,44	-0,07)	-0,13	**	(-0,27	0,01)

N: 13,129 observations from 370 units - each unit observed on average 36 months * = p < 0.05, ** = p < 0.01, *** = p < 0.001 Multilevel Poisson modelling with random intercept at unit and reginal health trust level

Table 3 displays the results from the multilevel model with propensity score matching. Across all units, we see in model 7 a significant reduction in total sickness absence in the first-year post-intervention (coef = -0.02, *p* < 0.05), but not in the second year (coef = -0.03, *p* > 0.05). When we examine long-term sickness absence in model 8, there was a significant reduction in the first (coef = -0.12, *p* < 0.001) and second (coef = -0.16, *p* < 0.001) year after the intervention.

**Table 3:** Multilevel Poisson regression^1^ with propensity score matched control groups

	All sickness absence				Long-term sickness absence (≥17 days)			
	M7		95% CI		M8		95% CI	
Matched controll unit	ref.				ref.			
Intervention unit	-0,23	**	(-0,37	-0,09)	-0,38	***	(-0,48	-0,28)
**Development in all units**	ref				ref			
First year	-0,02	*	(-0,04	0,00)	-0,12	***	(-0,14	-0,10)
Second year	-0,03		(-0,06	0,00)	-0,16	***	(-0,20	-0,12)
**Uniq development in intervention units**								
Intervention X First year	0,00		(-0,03	0,04)	0,02		(-0,03	0,07)
Intervention X Second year	0,01		(-0,05	0,08)	0,01		(-0,08	0,10)

N: 13,129 observations from 370 units - each unit observed on average 36 months * = p < 0.05, ** = p < 0.01, *** = p < 0.001 With random intercept at unit and reginal health trust level

Upon examining the unique development in the intervention units (i.e. Intervention * first year’ and ‘Intervention * second year), we found that the intervention units did not exhibit a significantly larger decrease in total (Model 7) or long-term sickness absence (Model 8) compared to the control units in either the first or second year.

Due to the significant differences between the intervention units and the matched control units in total absence prior to the intervention, we stress-tested the analyses with a second propensity score matching; matching units on total absence in 2020, regional health trust, and size (but not long-term absence). Results are presented in Appendix A. In the stress-test units were not significantly different on total absence prior to the intervention. In congruence with the main analyses the results of the stress-test did not support an effect of the intervention.

Due to a strong focus on tailoring, there is also a substantial heterogeneity in the details of the intervention. Through the framework “where the shoe pinched” each unit have uncovered different challenges and chosen different measures to address their specific challenges. As a result, we can expect the main effect of the intervention to be heterogeneous. In a random intercept model we assume the effect of the intervention to be the same for alle units, we can relax this by adding an additional random coefficient [38]. In Table 4 we repeat the analysis from Table 3 with a random intercept (M9 and M11), however for a more parsimoniously model we do not distinguish between the first and second year after the intervention. We then add a random slope to the model (M10 and M12). The random slope models allowed for each intervention unit to have their own unique development after the intervention (i.e. the random slope). For total absence the results are the same after adding a random slope (M10). There is a significant decline in total absence over time, but this decline is not significantly different between the intervention and control group. For long-term absence we do find a different results after adding a random slope. There is a significant decline in in long-term absence and this decline is significantly larger in the intervention units compared to control units. Noticeably, the coefficient for the random slope is also relatively substantial – indicating that the intervention has likely been successful in some units – and not in others.

**Table 4:** Multilevel Poisson regression^1^ with propensity score matched control groups and random slope

	All sickness absence								Long-term sickness absence (≥17 days)							
	Without Random slope				With Random slope				Without Random slope				With Random slope			
	M9		95% CI		M10		95% CI		M11		95% CI		M12		95% CI	
Matched controll unit	ref.				ref.				ref.				ref.			
Intervention unit	-0,23	***	(-0,37	-0,09)	-0,18	*	(-0,32	-0,04)	-0,38	***	(-0,48	-0,28)	-0,30	***	(-0,40	-0,20)
Development in all units	-0,02	***	(-0,04	0,00)	-0,02	*	(-0,04	-0,01)	-0,13	***	(-0,15	-0,11)	-0,13	***	(-0,15	-0,11)
Unique development in intervention units	0,01		(-0,03	0,04)	0,04		(-0,03	0,11)	0,02		(-0,03	0,06)	-0,13	*	(-0,25	-0,02)
constant	0,99		(0,14	1,83)	0,96	*	(0,11	1,81)	0,63	***	(0,36	0,90)	0,12	***	(0,45	0,92)
*Regional health trust*																
Random intercept	0,76		(0,18	3,12)	0,76		(0,18	3,14)	0,05		(0,02	0,31)	0,06		(0,01	0,24)
*Unit*																
Random intercept	1,81		(1,53	2,14)	1,94		(1,64	2,29)	0,14		(1,27	1,81)	1,35		(1,13	1,62)
Random slope: development in intervention units					0,06		(0,04	0,09)					0,26		(0,17	0,41)

N (observation): 13 129 N(units): 370 N(health trust): 4 * = p < 0.05, ** = p < 0.01, *** = p < 0.001 ^1^With random intercept at unit and reginal health trust level, and a random slope at unit level for unique development in intervention units

### Method 2 longitudinal survey

#### Descriptive

In Table 5, we present the descriptive statistics for all variables, categorized into pre- and post-intervention groups. Prior to the intervention, 71% of respondents reported taking sick leave, compared to 75% post-intervention. Regarding long-term sick leave, 12% reported taking it before the intervention, decreasing to 11% afterwards. Job satisfaction, on a scale of 1–7, was notably high, averaging 5.56 before and increasing to 5.81 after the intervention. In terms of general health, respondents rated their health at an average of 72 out of 100, both before and after the intervention.

**Table 5:** Descriptive statistics for survey data

	Intervention	
	Pre	Post
Sick leave (Yes - No)	0.71	0.75
Long-term sick leave (Yes - No)	0.12	0.11
Job satisfaction (1–7)	5.56	5.81
General health (0-100)	72	72
Controls		
Works evening or night	64%	71%
Women	89%	90%
Age		
30 or younger	24%	21%
31-40	20%	20%
41-50	23%	24%
51-60	23%	25%
Older than 60	10%	10%

Regarding control variables, 64% of respondents worked night or evening shifts prior to the intervention, which increased to 71% post-intervention. The demographic was predominantly female, with 89% women before the intervention and 90% afterwards. The age distribution varied: 24% were 30 years or younger before the intervention and 21% after; 20% were aged 31–40 years both before and after the intervention; 23% were in the 41–50 years age group before and 24% after; 23% were between 51 and 60 years before the intervention, increasing to 25% after; and 10% were over 60 years old at both times.

### Regressions

In Table 6, we present the analysis results of the survey data. For the continuous outcomes *job satisfaction* and *general health*, we employed multilevel linear regression. In these models, the coefficients represent the change in the outcome variable after the intervention is implemented. For the binary outcomes *sickness absence* and *long-term sickness absence* models 15, 16, 19 and 20, we employed multilevel logistic regression. In these models, the coefficients represent the change in the log-odds of the outcomes after the intervention is implemented. Model 13 shows a significant increase in job satisfaction post-intervention compared to two weeks prior to implementation (coef = 0.23, *P* < 0.05). This finding persists even after introducing control variables in model 17. However, no significant correlations were observed between the intervention’s implementation and other outcomes, including general health and sickness absence.

**Table 6:** Multilevel analysis of survey data

	Job satisfaction				General health			Sickness absence			Long-term sickness absence		
	M13				M14			M15			M16		
Intervention	0.23	*	(0.01	0.44)	-0.78	(-3.39	1.82)	0.24	(-0.29	0.77)	-0.08	(-0.67	0.51)
	M17				M18			M19			M20		
Intervention with controls	0.22	*	(0.01	0.42)	-0.59	(-2.85	1.67)	0.28	(-0.27	0.83)	-0.02	(-0.62	0.57)

* = *p* < 0.05, ** = *p* < 0.01, *** = *p* < 0.001 Controls include: age, sex and whether the respondent worked evening shifts or night shift

### Implementation prosses

Table 7 presents data on six dimensions of the implementation process, with the first five variables measured two weeks into the intervention (T2). In line with Randall et al. [35], variables are converted to a scale from 0 to 100. On average, employee involvement scored 61, although there was some variation with a standard deviation of 25. Specifically, 25% of respondents reported scores of 45 or lower, while another 25% reported scores of 83 or higher.

**Table 7:** Percentiles

	Mean	Std.dev.	25%	50%	75%
Employee involvement (T2)	61	25	45	61	83
Unit leader involvement (T2)	55	18	43	57	69
Union reresentative involvement (T2)	53	21	40	53	73
Safety delegate involvement (T2)	56	20	40	60	80
Collaboration - workgroup (T2)	60	14	53	60	75
Focus on the intervention (T3)	35	21	20	40	60

All variables are recoden on a scale from 0 to 100 N: ANDREAS LEGG INN HER

The involvement of unit leaders, union representatives, and safety delegates yielded similar results, with an average score between 53 and 56. The variation here showed a standard deviation between 18 and 20, with 25% of respondents scoring around 40–43 or lower, and another 25% scoring between 69 and 80 or higher. Collaboration within workgroups recorded mean score of 60, and exhibited less variation, with a standard deviation of 14 and 25th and 75th percentiles at 20 and 60 respectively.

The focus on the intervention measured one year later (T3) averaged 35 with a standard deviation of 21, with 25% scoring 20 or lower, and another 25% scoring 60 or higher.

In sum, while many respondents have scored high on aspects of the implementation process, the moderate to high standard deviations and the differences between the 25th and 75th percentiles indicate there are differences in experiences and perceptions among employees.

## Discussion

“Where the shoe pinches” is an intervention aimed at reducing sickness absence by finding and executing solutions to challenges in the work environment, through collaborations between the units’ leaders, union representatives, and safety delegates. The results of the intervention were mixed. There was a significant decrease in total and long-term sickness absence in the intervention units both in the first and second year after the intervention, measured with HR registries. However, we only found partial support for a significant larger decrease in long-term sickness absence in the intervention units compared to the control units. We found no support for a larger decrease in total absence in the intervention units.

In the subsample of units that also participated in the survey, we observed a significant positive association between the intervention and employee job satisfaction. However, no significant associations were found between the intervention and general health or self-reported sickness absence. Based on the findings we see a clear reduction in sickness absences following the intervention. However, we find mixed support for whether this reduction, or part of this reduction, can be attributed to the intervention. The units were selected due to high sickness absence, and the observed reduction might be due to regression towards the mean [39], suggesting a possible decrease in absence regardless of the intervention.

Furthermore, without data on the activities of control units, it’s uncertain whether the observed reductions in the control units were also due to other initiatives in those units. It is reasonable to assume that the control units would not remain passive in the face of high sickness absence.

In the workplace in general, prior studies have found that interventions targeting the work environment may reduce sickness absence (sources), and as Brady et al. (source) argued, the work environment is likely a crucial determinant of sickness absence within the healthcare sector. Our results only partially support this explanation. In line with Simmons et al. [8] there is still a clear lack of interventions in the healthcare sector that effectively reduces sickness absence.

“Where the shoe pinches” is one of a growing number of tailoring interventions [18–21, 23–25]. While the framework consists of general tools and steps, the challenges and measures taken to address them are specific for the individual units. When we evaluate the results across seventy-eight intervention units, we analyse the mean results of using the overall framework “where the shoe pinches” to address sickness absence. We do not analyse the specific effects of the multitude of different solutions discovered and implemented using the framework, nor the differences in how the overall framework is interpreted and practiced at the individual units. Prior studies have supported that such tailor interventions are generally more effective than non-tailoring intervention [24, 25]. Some on these interventions have computers tailoring specific details based on standardised data collection, other interventions, like “where the shoe pinched” is a more comprehensive subjective tailoring process – increasing the overall heterogeneity. Our results support a heterogeneous main effect of the intervention, indicating that the intervention have been effective in some units but not in other.

Finally, while the measures that are implemented are meant to vary, our results also showed differences in how the overall framework of the intervention was implemented within the different units.

A key element of the intervention was employee involvement, which is recognized as an important prerequisite for successful change implementation [40–43]. The results supported a general high level of employee involvement. We also saw higher involvement in our study compared to Randall [35] (61 in our study compared to 42). However, our numbers were measured shortly after the intervention was started, which may have increased employees experience of involvement compared to later in the process. In a qualitative review of the same intervention some employees did rase concerns regarding low involvement [28].

A second crucial aspect of the intervention is involvement from the leader. While the process facilitator facilitates the first phases of the intervention – it is a prerequisite that the leader, in collaboration with the union representative and safety delegate, takes ownership over the latter phases of the intervention. Leader involvement has also been highlighted as important for successful implementation in prior studies [40, 44–48]. We saw lower involvement compared to Randall et al. [35] (55 compared to 61). Challenges with leader involvement and insufficient collaboration between the leader, union representatives and safety delegate were also stressed in the qualitative process evaluation [28]. As argued by Fjeldbraaten and Wathne [28] leaders likely have multiple tasks to handle on top of the intervention. Furthermore, some units had limited collaboration between the leader, union representatives and safety delegate prior to the intervention – making a core premiss of the intervention harder to establish. On the other hand, this also meant that the intervention could have a positive effect in establishing or improving the general collaboration between the parties.

Finally, the results showed substantial variation in time spent on the intervention. This was also a recognized problem in the qualitative evaluation of the intervention process [28]. To some extent the COVID-19 pandemic was partially held responsible. As stated by one employee: ““I felt that when we were done with that dialog workshop, that was it. We never heard anything more. And then of course, Corona came and we put a lid on it.“» [28].

### Strengths and limitation

The current study has several strengths. We have used an HR-registry to measure both total and long-terms sickness absence. Consequently, the measure is more objective, and not subject to recall-bias. Using HR-registry also ensures full participation and no dropouts apart from when employees leave the units.

The study covers a three-year span providing good information on the immediate and more long-term results following the intervention. The study also follows a high number of intervention units across all Norwegian regional health trusts – providing more robust information on the overall results of the intervention approach, less susceptible to qualities in the individual units.

Ideally, interventions should be tested using randomized controlled trials [49]. As this was not possible, we have used propensity score matching to create a control group that was similar to the intervention groups prior to the intervention. We did however have less information on the units than optimal for propensity score matching. It would have been preferably with additional information on the composition of employees such as professions and gender. Furthermore, we only had absence levels one year prior to the first intervention. A single absence spell in Norway may be up to 365 days. Consequently, a few employees with long absence spells may alone results in high absence rates for a unit one year, dropping the next if employees return to work or their employment ends. If intervention units were selected based on stable high absence rate over a longer time span – intervention units may be less likely than control unit to “regress towards the mean”. If this is the case the results of the intervention may be underestimated.

One strength of the study was that we were able to supplement the HR-registry with a longitudinal survey design in a subsample of units, investigating if a reduction in absence could be explained by improvements in employee health and job satisfaction. However, a low number of units limited the analyses possible, and made it difficult to interpret non-sig finings.

During the study period – both the implementation and sickness absence levels were affected by the COVID-19 pandemic. The use of matched control groups will have amended COVID-19 related fluctuations in absence levels. However, we cannot exclude that the implementation of the intervention would have been more successful under different circumstances. Both the pandemic restrictions and increased hospital staff demands made organizing intervention activities, like scheduling project meetings and involving external process facilitators, more challenging. Additionally, unit leaders might have allocated more time to the intervention if not for the heightened operational demands during COVID-19. Therefore, we cannot dismiss the possibility that the implementation and effectiveness of the intervention would have been better in a post-COVID setting.

One important potential bias in our study is employee turnover, which may influence the observed changes in sickness absence. If unhappy or unhealthy employees leave, unit sickness absence might decrease, potentially explaining the reduction in absence in the fixed effects analyses. This bias is likely mitigated in the multilevel analyses using HR registry data with a control group, since both intervention and control groups, selected for high absence, could experience similar turnover effects due to poor work environments. However, specific circumstances, like increased awareness from the intervention “where the shoe pinches” possibly urging a reduction in absences, might affect decisions differently across groups.

The survey results may further be impacted by bias in who responded at T1 and who responded at T3. Although Wolke et al. [50] suggest that high dropout rates minimally impact study validity, and Hellevik [51] argues that even substantial nonresponse does not necessarily bias most results, Beller et al. [38] note that employee health can affect participation. This is particularly relevant when examining changes in health-related outcomes, emphasizing the need to consider potential biases due to varying response rates. A strength of the current study is the combination of survey data with HR registry data reducing the risk of wrongfully concluding based on survey data alone. Regardless, we cannot exclude biases such as if improved job satisfaction is caused by higher response rate at T3 among satisfied employees, or that the relationship with general health is masked by a higher response rate at T1 among employees with chronic conditions.

## Conclusion

There is a stark need for research on effective interventions to reduce sickness absence in the health care sector. “Where the shoe pinches” provides a methodological framework for reducing sickness absence by addressing challenges in the work environment. The intervention has a strong focus on tailoring, employee involvement, and collaboration between the leader, union representative and safety delegate, through a four-stage process. The results of the intervention were mixed. There was a decrease in sickness absence in the intervention units after the intervention, despite a general increase in absence in the sector during the same period. However, compared to a matched control groups we did not see a significant larger decrease in total absence in the intervention units. We did find partial support for a larger decrease in long-term absence in the intervention units, and a significant increase in job satisfaction after the implementation of the intervention. Consequently, further exploration is warranted to discern the precise mechanisms underlying the intervention’s impact and to refine strategies for effectively managing sickness absence within healthcare organizations.

## Data Availability

The health trusts are the owners of all data from the HR registries and should be contacted for access. Anonymous survey data may be made available upon reasonable request to the corresponding author.
